# Traditional Chinese medicine Jia Wei Gui Pi Tang improves behavioural and psychological symptoms of dementia and favourable positive emotions in patients

**DOI:** 10.1111/psyg.12962

**Published:** 2023-04-02

**Authors:** Tatsuya Nogami, Koh Iwasaki, Hideo Kimura, Toru Higashi, Makoto Arai, James P. Butler, Masahiko Fujii, Hidetada Sasaki

**Affiliations:** ^1^ Department of Kampo Medicine Tokai University School of Medicine Isehara City Japan; ^2^ Ayumino Clinic Ishinomaki City Japan; ^3^ Department of Kampo Medicine Sakurajyuji Hospital Fukuoka City Japan; ^4^ Department of Psychiatry Ainohanazono Hospital Ibaragi City Japan; ^5^ Department of Environmental Health Harvard TH Chan School of Public Health Boston Massachusetts USA; ^6^ Brigham and Women's Hospital Harvard Medical School Boston Massachusetts USA; ^7^ Sendai‐Tomizawa Hospital Sendai City Japan

**Keywords:** anxiety, behavioural and psychological symptoms of dementia (BPSD), favourable positive emotions, Jia Wei Gui Pi Tang, Kampo, traditional Chinese medicine

## Abstract

**Background:**

Behavioural and psychological symptoms of dementia (BPSD) are challenging to manage, leading to caregiver burden and often to subsequent transfer of patients to a nursing home or psychiatric hospital for treatment. Eliciting favourable positive emotions should be an important goal in the treatment of negative emotions associated with BPSD. To date, no data have indicated that antipsychotic medications can improve positive emotions. BPSD are known to be associated with anxiety in patients with dementia. The traditional Chinese medicine Jia Wei Gui Pi Tang is officially indicated and approved for anxiety treatment in Japan.

**Methods:**

Here, we performed a multicentre, randomised, observer‐blind control study of the effect of Jia Wei Gui Pi Tang on BPSD in Alzheimer's disease (AD) patients. Patients with AD or AD with cerebral vascular disease were randomly divided into the Jia Wei Gui Pi Tang treatment group and the control group that received no traditional Chinese medicine. BPSD were scored using the Neuropsychiatric Inventory Nursing Home Version (NPI‐NH) and by favourable positive emotions using the Delightful Emotional Index (DEI).

**Results:**

A total of 63 participants (18 male and 45 female; mean age: 83.3 ± 6.0 years) were included in the study. Changes in NPI‐NH scores differed significantly between the two groups (one‐way analysis of variance, *P* < 0.001). Within the treatment group, there was a significant improvement in the NPI‐NH score from 29.8 ± 17.3 at baseline to 13.2 ± 9.4 at the endpoint (paired *t*‐test, *P* < 0.001), whereas there was no statistically significant change in the control group. Changes in DEI scores differed significantly between the two groups. Within the treatment group, there was a significant improvement in the DEI score from 24.3 ± 23.0 at baseline to 32.5 ± 21.2 at the endpoint (paired *t*‐test, *P* = 0.001), whereas there was no statistically significant change in the control group.

**Conclusion:**

The traditional Chinese medicine Jia Wei Gui Pi Tang significantly improved both BPSD and positive emotions.

## INTRODUCTION

Behavioural and psychological symptoms of dementia (BPSD) are challenging to manage, leading to caregiver burden and often to subsequent transfer of patients to a nursing home or psychiatric hospital for treatment of dementia.^1^, ^2^ Following our report in 2005 that the traditional Chinese medicine (TCM) Yigan San (抑肝散) improved BPSD,^3^ over 400 papers have been published in the English language on the effects and mechanisms of Yigan San,^4^, ^5^ which is currently used widely for treatment of BPSD.

In addition, since 2005, many antipsychotics have been developed.^6^, ^7^ These drugs improve BPSD, but there are no data to suggest that they improve favourable positive emotions, such as manifested by the exchange of appropriate greetings, facial expressiveness, showing interest or delight in something, and carrying on conversations. Furthermore, in April 2005, the US Food and Drug Administration issued a public health advisory to alert health care providers, patients, and patient caregivers to new safety information concerning the unapproved use of atypical antipsychotic drugs.^8^

Recently, we reported that theatre performance can both improve BPSD and favour positive emotions.^9^ However, such a treatment program requires the availability of well‐trained actors, and the cost can place a prohibitively high burden on many hospitals.

Interestingly, in our daily practice, we have found that the TCM Jia Wei Gui Pi Tang (加味帰脾湯) appears to improve both BPSD and positive emotions. This medication is a mixture of herbs, as detailed in Section 2.3 below. Jia Wei Gui Pi Tang has four official indications for treatment in Japan: anaemia, insomnia, anxiety, and neurosis. Jia Wei Gui Pi Tang is often used to treat anxiety in daily practice, but its effectiveness for treating anxiety in older patients with dementia has not been studied to date. BPSD are causally associated with anxiety in patients with dementia.^10^, ^11^, ^12^ There are some traditional textbooks^13^ that reference the historical use of Jia Wei Gui Pi Tang based on the book *Xue‐shi‐yi‐an* (薛氏医案) by Xue Ji (薛己),^14^ who was active in the 15th and 16th centuries, which described the efficacy of this TCM in improving symptoms such as insomnia, fever, sweating, anxiety, abnormal activity, amnesia, and appetite loss. These conditions correlate well with BPSD. As such, Jia Wei Gui Pi Tang is a natural candidate for treating BPSD through its effect on anxiety. However, its efficacy in treating BPSD in Alzheimer's disease (AD) patients has not been reported previously.

Therefore, we report herein an observer‐blind randomised control trial to assess the efficacy of this TCM for both treating BPSD and enhancing positive emotions in patients with dementia. For the benefit of a wider readership, we include the transliteration of Jia Wei Gui Pi Tang not only in Chinese characters as given above, but also as romanised in Japanese (Kamikihito) and Korean (Kami‐Guibi‐Tang).

## METHODS

### Study design and overview

The present study (UMIN000045650) was a multicentre, randomised, observer‐blind control study on patients with AD and mixed type dementia (AD with cerebral vascular disease). Participants were randomly assigned in an observer‐blind manner to one of two groups: a treatment group that received Jia Wei Gui Pi Tang treatment and a control group that did not receive any TCM. Participants were then followed up and their BPSD, favourable positive emotions, and cognitive function were observed.

The present study was carried out in compliance with the Helsinki Declaration, the clinical research law of Japan, and the study protocol. Four institutions throughout Japan participated in the trial after obtaining approval from their local institutional review boards. Approval numbers from the respective Ethics Committees were: Sendai‐Tomizawa Hospital, 21‐10‐1; Natori‐Kumanodo Hospital, 21‐001; Ainohanazono Hospital, 21‐001; Sakurajyuji Hospital, SJF‐2021022001. Written informed consent to participate in this study was obtained from each participant or their guardian. The data were collected from October 2021 to January 2022.

### Participants and group assignments

All participants were recruited from four institutions for long‐term care located in the prefectures of Fukuoka, Osaka, and Miyagi in Japan. A total of 63 patients were diagnosed with dementia according to the Diagnostic and Statistical Manual of Mental Disorders, 5th ed. (DSM‐V) criteria. The physical condition of all patients had been stable for the past year. At baseline, all patients underwent a uniform evaluation, which included a medical history, physical and neurological examination, and brain computed tomography scan, as well as the Mini‐Mental State Examination (MMSE)^15^ to assess cognitive function. BPSD were assessed and evaluated using the Neuropsychiatric Inventory Nursing Home Version (NPI‐NH, Japanese edition; Micron Co. Ltd., Aichi, Japan).^16^ Individuals diagnosed with AD or AD with vascular cognitive disorder according to the DSM‐V were eligible if they met all the following inclusion criteria: (i) age 65–100 years; (ii) total NPI‐NH score of >3; (iii) positive NPI‐NH subcategory scores for “anxiety” or “problems in night‐time”; and (iv) MMSE score of <25. Individuals were excluded if they had experienced a major depressive or bipolar disorder episode within the previous year, had been on antidepressants or other herbal medicines, or had a malignant tumour or other life‐threatening disease within the previous 2 years. All patients taking antidepressants were excluded; however, use of cholinesterase inhibitors and memantine for at least 3 months, anxiolytics, hypnotics, and anticonvulsants was permitted.

#### Randomisation

Patients were randomised (1:1 ratio) to each group using the stratified method, with balancing of the arms based on the NPI‐NH scores. Computer‐generated random numbers were used for stratified randomisation.

#### Blinding

The study was conducted in open‐label fashion with no blinding for the patients or researchers, but observers were assigned by caregivers who were not informed of the patient's medication regimen at each facility.

### Study medication protocol

The active study medication Jia Wei Gui Pi Tang (Tsumura Kamikihito Extract Granules for Ethical Use, TJ‐137) was obtained from Tsumura & Co. (Tokyo, Japan) and was administered orally three times a day (2.5 g each, 7.5 g/day) over 28 days in the treatment group. This TCM contains 14 herbs: *Astragali Radix*, *Bupleuri Radix*, *Zizyphi Semen*, *Atractylodis Lanceae Rhizoma*, *Ginseng Radix, Poria*, *Polygalae Radix*, *Gardeniae Fructus*, *Zizyphi Fructus*, *Angelicae Radix*, *Glycyrrhizae Radix*, *Zingiberis Rhizoma*, *Saussureae Radix*, and *Longan Arillus* (Table 1). Detailed information on this TCM is available on the website STORK (Standards of Reporting Kampo Products; http://mpdb.nibiohn.go.jp/stork/), and information on the production and quality control systems for Kampo products is available on the Tsumura website (https://www.tsumura.co.jp/english/kampo/07.html). The control group received no TCM, and the treatment group received no TCM other than Jia Wei Gui Pi Tang. Other medications such as those listed in Section 2.2 above were continued, and additional non‐TCM medications were permitted if deemed medically necessary by the attending physician. Environmental adjustment and caregiver support were provided to all participants.^9^

**Table 1:** Herbs present in Jia Wei Gui Pi Tang used in this study

Herb name	Primitive plants
*Astragali Radix*	*Astragalus membranaceus* Bunge
*Bupleuri Radix*	*Bupleurum falcatum* Linne
*Zizyphi Semen*	*Ziziphus jujuba* Mill. var. *spinosa* Hu ex H. F. Chou
*Atractylodis Lanceae Rhizoma*	*Atractylodes lancea* De Candolle
*Ginseng Radix*	*Panax ginseng* C. A. Meyer
*Poria*	*Wolfporia cocos* Ryvarden et Gilbertson
*Polygalae Radix*	*Polygala tenuifolia* Willdenow
*Gardeniae Fructus*	*Gardenia jasminoides* J. Ellis
*Zizyphi Fructus*	*Ziziphus jujuba var. inermis* Rehder
*Angelicae Radix*	*Angelica acutiloba* Kitagawa
*Glycyrrhizae Radix*	*Glycyrrhiza uralensis* Fischer et De Candolle
*Zingiberis Rhizoma*	*Zingiber officinale* Roscoe
*Saussureae Radix*	*Saussurea lappa* C. B. Clarke
*Longan Arillus*	*Euphoria longana* Lamarck

### Outcome measures

The main outcome was total changes in the NPI‐NH scores for BPSD as measured by a comparison of assessments undertaken before and after the 28‐day study.^16^ The major secondary outcome was change in the Delightful Emotional Index (DEI) reflecting favourable positive emotions,^17^ and another secondary outcome was change in the MMSE scores in cognitive function. The DEI is scored using the following 10 different items: (i) Ability to exchange greetings appropriately; (ii) Exhibit facial expressiveness; (iii) Show interested in something; (iv) Carry on a conversation; (v) Show delight with something; (vi) Express one's gratitude; (vii) Respond to applause; (viii) Show anxiety about something; (ix) Have a sense of humour; and (x) Show the ability to differentiate between beauty and ugliness. In each item, four different degrees of reactions were recorded using the following 4‐point scale: no response = 0, mild = 1, moderate = 2, and normal = 3. In addition, four different degrees of temporal frequency were recorded also using a 4‐point scale: none = 0, once every few days = 1, a few times per day = 2, and almost every day = 3 points. The score for each item is the product of the response score and the frequency score, thus spanning 0–9 for each item. Finally, each patient was given a cumulative score given by the sum of these 10 items, thus spanning a final range of 0–90.

Changes in monthly biochemical profiles, including liver and renal function and serum potassium, were measured both before and after the study period and were considered potential secondary outcomes.

### Statistical analysis

A target sample size of 60 patients, with 30 patients in each group, was planned to provide the study with 80% power for an effect size of 8 points on the mean NPI‐NH scores before and after the study period in the treatment group. The control group was evaluated with a two‐sided type I error of 0.05 considering the minimum clinically important difference (MCID) of NPI‐NH (reported as 8.0 points).^18^ To compensate for potential loss during follow‐up and patient exclusion, we enrolled a few additional individuals, resulting in *n* = 33 in the treatment group and 30 in the control group. This has a negligible effect on the calculated 80% power cited above.

The analysis set for the efficacy evaluation included patients who were eligible and had started treatment. All participants who took trial medications were included in the safety analyses. Statistical analysis was performed using BellCurve for Excel software (ver. 3.21, Social Survey Research Information Co., Ltd., Tokyo, Japan). Baseline comparisons of group characteristics were conducted using the independent samples *t*‐test for continuous variables and the chi‐squared test for categorical variables. Mean and standard deviation for all parameters were determined at baseline and at the endpoint of the study. Comparisons between the treatment and control groups were performed by one‐way analysis of variance (anova) using the two‐tailed Welch test (for intergroup differences). Changes within groups from before to after the trial period were compared using a paired *t*‐test (intragroup difference). *P* values of <0.05 were considered significant.

## RESULTS

**Figure 1:**
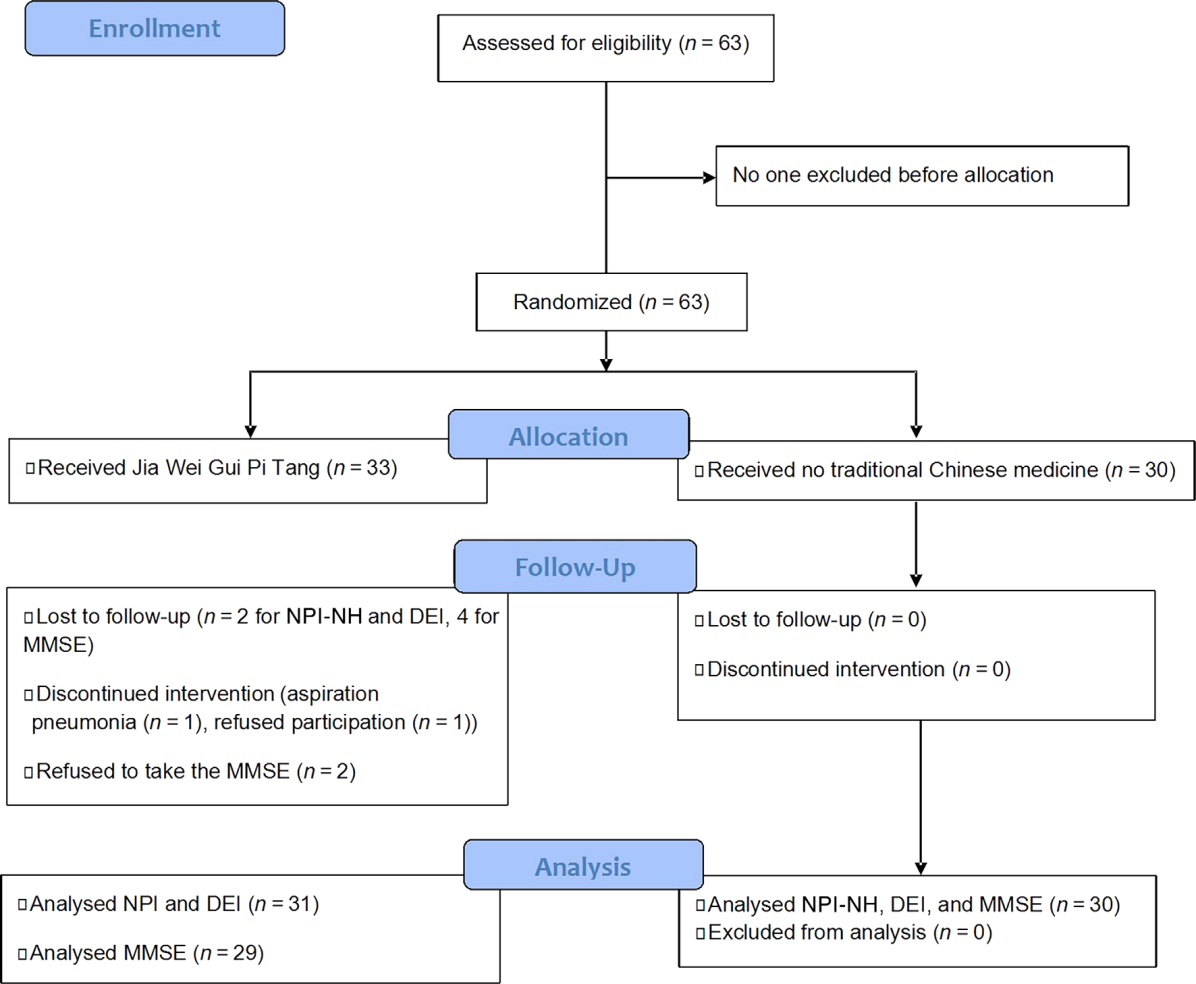
The flow diagram of the study. DEI, Delightful Emotional Index; MMSE, Mini‐Mental State Examination; NPI‐NH, Neuropsychiatric Inventory Nursing Home Version, Japanese edition.

A total of 63 participants (18 male and 45 female participants; mean age: 83.3 ± 6.0 years) were included in the study. The flow diagram is shown in Fig. 1. The demographic characteristics and NPI‐NH, DEI, and MMSE scores in each group at baseline are shown in Table 2. There was no significant difference between the groups in terms of age, sex, NPI‐NH score, or MMSE score. However, there was a statistically significant difference in the DEI scores between the groups at baseline. No laboratory blood chemical data, including liver and renal function and electrodes, were significantly different between groups.

**Table 2:** Demographic characteristics, NPI‐NH scores, DEI scores, and MMSE scores in each group at baseline

Group characters at baseline	Jia Wei Gui Pi Tang	Control	*P* *
*N*	33	30	
Sex			
Male	10	8	
Female	23	22	0.750
Age	82.5 ± 6.6	84 ± 5.1	0.226
NPI‐NH score	28.7 ± 17.5	29 ± 20.4	0.950
DEI score	23.3 ± 22.9	37 ± 27.3	0.037*
MMSE score	11.4 ± 7.4	13 ± 6.0	0.241

* Statistical significance (*P* < 0.05).

Abbreviation: DEI, Delightful Emotional Index; MMSE, Mini‐Mental State Examination; NPI‐NH, Neuropsychiatric Inventory Nursing Home Version, Japanese edition.

### Changes in NPI‐NH, DEI, and MMSE scores

Two patients in the treatment group dropped out of the study due to aspiration pneumonia and wished to discontinue participation, leaving 31 participants in the treatment group and 30 participants in the control group. All participants completed the NPI‐NH score and DEI score assessments before and after the observation period, but two participants in the treatment group refused to take the MMSE test at the endpoint. Therefore, MMSE data were analysed for 29 participants in the treatment group and 30 participants in the control group.

**Figure 2:**
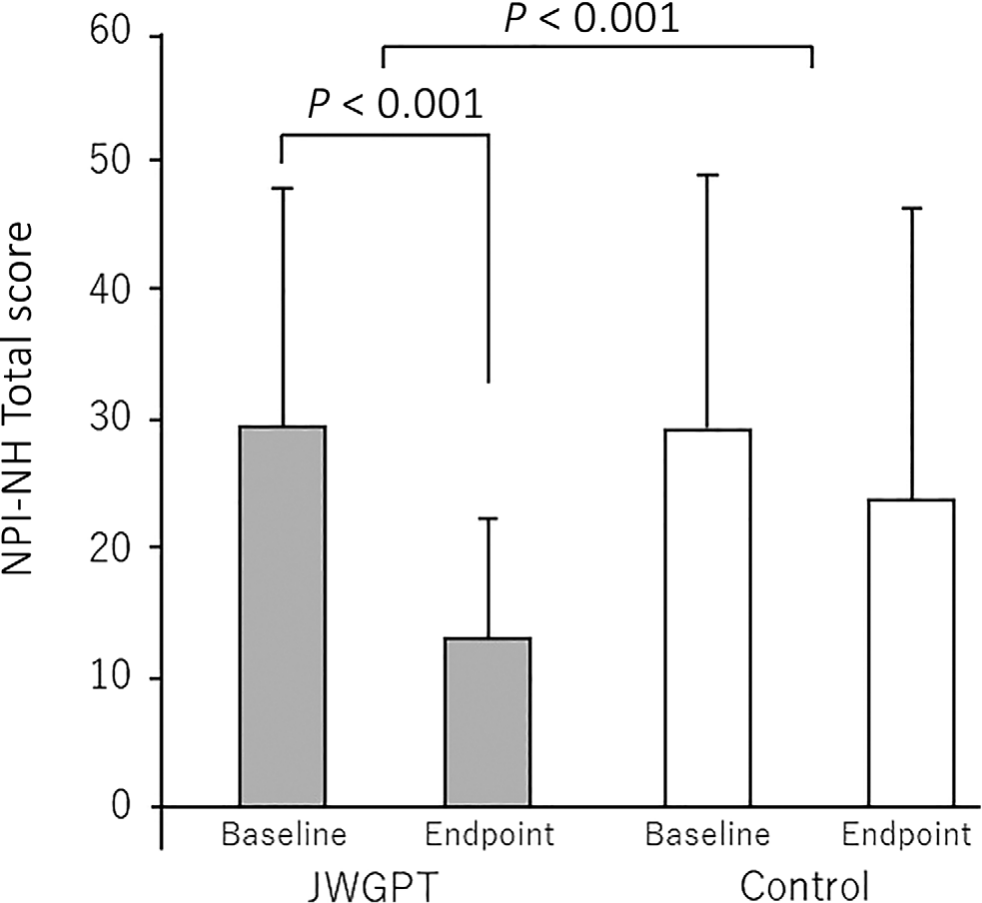
Changes in the NPI‐NH total score. One‐way analysis of variance shows a significant difference between the groups (*P* < 0.001). In the treatment group, the NPI‐NH total score improved significantly from 29.8 ± 17.3 to 13.2 ± 9.4 (paired *t*‐test; *P* < 0.001), while in the control group it changed from 29.0 ± 20.4 to 24.6 ± 23.1, with no statistical significance (paired *t*‐test; *P* = 0.056). JWGPT, Jia Wei Gui Pi Tang; NPI‐NH, Neuropsychiatric Inventory Nursing Home Version, Japanese edition.

**Figure 3:**
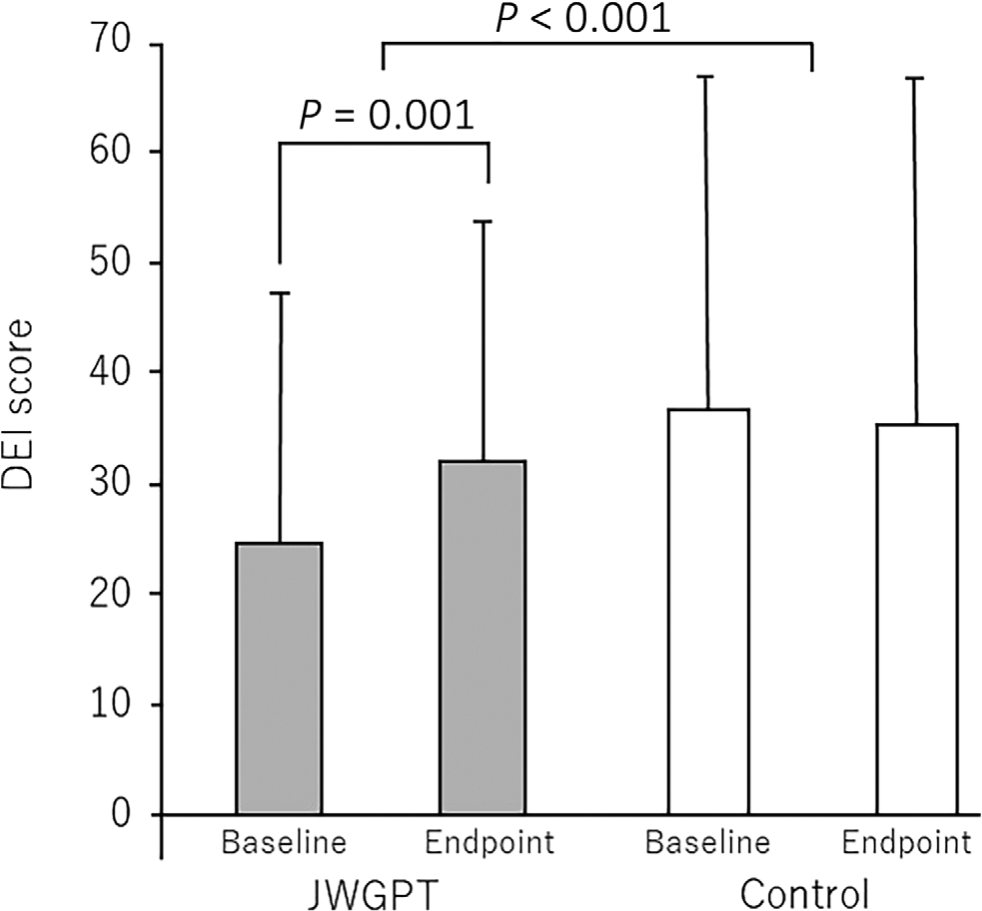
Changes in the DEI dementia score. One‐way analysis of variance shows a significant difference between the groups (*P* < 0.001). In the treatment group, the DEI score improved significantly from 24.4 ± 23.2 to 32.5 ± 21.3 (paired *t*‐test; *P* < 0.001), while in the control group it changed from 37.0 ± 27.3 to 34.6 ± 28.8 with no statistical significance (paired *t*‐test; *P* = 0.105). DEI, Delightful Emotional Index; JWGPT, Jia Wei Gui Pi Tang.

Changes in the NPI‐NH scores differed significantly between the two groups (one‐way anova, *P* < 0.001). Within the treatment group, there was a significant improvement in the NPI‐NH score between baseline and the endpoint, from 29.8 ± 17.3 points to 13.2 ± 9.4 points (paired *t*‐test, *P* < 0.001), whereas there was no statistically significant change in the control group (Fig. 2). Changes in the DEI scores differed significantly between the two groups (one‐way anova, *P* < 0.001). Within the treatment group, there was a significant improvement in the DEI score between baseline and the endpoint, from 24.3 ± 23.0 points to 32.5 ± 21.2 points (paired *t*‐test, *P* = 0.001), whereas there was no statistically significant change in the control group (Fig. 3). Changes in the MMSE scores did not differ significantly between the two groups (one‐way anova, *P* = 0.479), and there was also no significant difference between the MMSE scores at baseline and at the endpoint in either group (Table 3).

**Table 3:** Changes in NPI‐NH, DEI, and MMSE scores

Group characters at baseline	Jia Wei Gui Pi Tang Group (*N* = 31)			Control Group (*N* = 30)			Intergroup difference
	Baseline	Endpoint	Intragroup difference	Baseline	Endpoint	Intragroup difference	
NPI score	29.8 ± 17.3	13.2 ± 9.4	*P* < 0.001*	29.0 ± 20.4	24.6 ± 23.1	*P* = 0.056	*P* < 0.001*
DEI score	24.3 ± 23.0	32.5 ± 21.2	*P* = 0.001*	37.0 ± 27.3	34.6 ± 28.8	*P* = 0.105	*P* < 0.001*
MMSE score^†^	11.7 ± 7.5	11.4 ± 8.0	*P* = 0.527	13.4 ± 5.6	13.5 ± 6.1	*P* = 0.707	*P* = 0.479

^†^ MMSE scores at the study endpoint were not available in two cases. Thus, there were only 29 cases in the Jia Wei Gui Pi Tang group for this measure.

* Statistical significance (*P* < 0.05).

Abbreviation: DEI, Delightful Emotional Index; MMSE, Mini‐Mental State Examination; NPI‐NH, Neuropsychiatric Inventory Nursing Home Version, Japanese edition.

### Changes in NPI‐NH subcategory scores

Statistically significant differences (one‐way anova) were observed between the two groups for five NPI‐NH subcategory scores: agitation/aggression, dysphoria, anxiety, disinhibition, and irritability/lability. In the treatment group, scores had improved significantly between baseline and the endpoint in eight NPI‐NH subcategories: delusions, agitation/aggression, dysphoria, anxiety, disinhibition, irritability/lability, aberrant motor activity, and night‐time disturbance. In the control group, only one NPI‐NH subcategory score, aberrant motor activity, was significantly improved at the endpoint (Table 4).

**Table 4:** Changes in NPI‐NH subcategory scores

Group characters at baseline	Jia Wei Gui Pi Tang group (*N* = 31)			Control group (*N* = 30)			Intergroup difference
	Baseline	Endpoint	Intragroup difference	Baseline	Endpoint	Intragroup difference	
Delusions	1.5 ± 3.4	0.7 ± 2.4	*P* = 0.021*	1.7 ± 2.6	1.1 ± 2.2	*P* = 0.142	*P* = 0.614
Hallucinations	1.1 ± 2.6	0.6 ± 1.6	*P* = 0.083	1.0 ± 2.1	0.8 ± 1.4	*P* = 0.636	*P* = 0.475
Agitation/aggression	4.6 ± 3.5	1.5 ± 1.8	*P* < 0.001**	4.2 ± 4.0	3.4 ± 3.5	*P* = 0.259	*P* = 0.012*
Dysphoria	2.4 ± 2.5	1.4 ± 1.9	*P* = 0.018*	2.1 ± 2.3	2.4 ± 2.9	*P* = 0.408	*P* = 0.016*
Anxiety	3.6 ± 3.4	1.4 ± 1.7	*P* < 0.001**	2.4 ± 2.8	2.3 ± 2.9	*P* = 0.640	*P* < 0.001**
Euphoria	0.2 ± 0.6	0.1 ± 0.3	*P* = 0.325	0.5 ± 1.0	0.5 ± 1.0	*P* = 0.662	*P* = 0.293
Apathy	3.2 ± 2.7	2.6 ± 2.1	*P* = 0.071	4.2 ± 4.0	4.0 ± 3.5	*P* = 0.832	*P* = 0.524
Disinhibition	2.9 ± 3.5	0.9 ± 1.3	*P* < 0.001**	2.2 ± 3.9	1.9 ± 3.5	*P* = 0.464	*P* = 0.010*
irritability/lability	4.9 ± 3.6	1.7 ± 2.0	*P* < 0.001**	3.9 ± 4.1	3.0 ± 3.7	*P* = 0.072	*P* = 0.003**
Aberrant motor activity	2.8 ± 3.6	1.8 ± 2.8	*P* = 0.045*	3.3 ± 4.1	1.8 ± 2.5	*P* = 0.032*	*P* = 0.605
Night‐time disturbance	2.2 ± 2.9	0.7 ± 1.5	*P* = 0.006**	2.6 ± 3.5	2.2 ± 3.2	*P* = 0.610	*P* = 0.176
Eating disturbance	0.4 ± 2.2	0.0 ± 0.0	*P* = 0.288	1.0 ± 2.3	1.1 ± 2.0	*P* = 0.677	*P* = 0.294

* *P* < 0.05;

** *P* < 0.01.

Abbreviation: NPI‐NH, Neuropsychiatric Inventory Nursing Home Version, Japanese edition.

## DISCUSSION

In the present study, both BPSD and favourable positive emotions were improved following the use of Jia Wei Gui Pi Tang. Use of this TCM markedly improved the DEI scores, while there was no significant improvement in the DEI scores in the control group. The total NPI‐NH score improved by 16.6 points, from 29.8 ± 17.3 to 13.2 ± 9.4, in the treatment group, and by 4.4 points, from 29.0 ± 20.4 to 24.6 ± 23.1, in the control group, resulting in a difference of 12.2 in improved points; thus, the difference between the two groups exceeded the MCID of the NPI‐NH, previously reported as 8.0.^18^

Lin *et al*.^19^ previously suggested the efficacy of Jia Wei Gui Pi Tang in treating patients with anxiety. BPSD are also strongly related to anxiety.^10^, ^11^, ^12^ In the present study, we showed that Jia Wei Gui Pi Tang improved agitation, dysphoria, anxiety, disinhibition, and irritability associated with BPSD in AD patients.

Some studies have reported that Jia Wei Gui Pi Tang improves dementia. Shin *et al*.^20^ reported that this TCM ameliorated mild cognitive impairment in a double‐blinded randomised control study. In animal models, Watari *et al*.^21^ reported that it improved amyloid‐beta‐induced tau phosphorylation and axonal atrophy. Moreover, it improved object‐recognition memory deficit in an AD model mouse. Restoration of degenerated axons and synapses may be associated with memory facilitation by this TCM.^22^ Araki *et al*.^23^ reported that Jia Wei Gui Pi Tang ameliorated sickness behaviour in mice inoculated with murine colon 26 adenocarcinoma cells. Furthermore, Adachi *et al*.^24^ reported that this TCM reduced depressive‐like behaviours and hippocampus neurogenesis in chronic restraint stressed rats.

In the present study, the MMSE score remained statistically unchanged, implying that Jia Wei Gui Pi Tang does not contribute significantly to a decline in cognitive functioning.

Finally, many reports have shown that the herbs contained in Jia Wei Gui Pi Tang improve anxiety,^24^ depression,^25^, ^26^, ^27^, ^28^, ^29^, ^30^, ^31^, ^32^, ^33^, ^34^ and hallucinations.^26^ Therefore, we believe that Jia Wei Gui Pi Tang may improve favourable positive emotions.

The present study had some limitations. First, it was not a placebo‐controlled double‐blind study, as an appropriate placebo trial was not implemented. Second, the MMSE is not sensitive enough to assess small cognitive changes within the short span of 28 days. Third, there was a statistically significant difference in the DEI scores between the control and treatment groups at baseline (Table 2). Since the participants in each group were divided randomly, this was a surprising finding. However, it is noteworthy that the *P*‐value was near the threshold of significance, a phenomenon not shared by the other measures. Fourth, we calculated our sample size based only on the NPI‐NH. Fifth, the DEI has not been statistically validated, although it has already been applied in some studies (e.g., though unreported, by the authors of,^17^ based on personal communication).

More research will be needed to confirm the positive effects of Jia Wei Gui Pi Tang, particularly in terms of the improvement in the DEI. Finally, while not strictly a limitation, we noted that *Glychirrhizae Radix* can cause hypokalemia.^35^ Therefore, serum potassium concentration should be checked at least once a month. *Gardeniae Fructus* may cause idiopathic mesenteric phlebosclerosis over a long time, spanning years or decades^36^; however, this is not likely to be important in the treatment of BPSD.

The TCM Jia Wei Gui Pi Tang improved both BPSD and positive emotions, without worsening cognitive function. Therefore, it has the potential not only to address BPSD, but importantly, also to improve the emotional dimension of delight, a key feature in the support and care of those with dementia.

## AUTHOR CONTRIBUTIONS

TN, KI, and HS designed the study. TN and KI analysed the data and wrote the manuscript. KI, HK, TH, and HS collected the data. MA and MF supervised this study. JB advised on the research and proofread the English. All authors read and approved the final manuscript.

## DISCLOSURE

Tatsuya Nogami and Makoto Arai received lecture fees from the Department of Kampo Medicine, Tokai University, School of Medicine, to which they belong, and received a research grant from Tsumura & Co. that was not related to this study.

## FUNDING INFORMATION

The study did not receive funding from any external sources. The cost of the study was covered by the Sendai Tomizawa Hospital's own funds.

## Data Availability

The data that support the findings of this study are available from the corresponding author upon reasonable request.
